# Role of foetal kidney size on kidney function in childhood: the born in bradford cohort renal study

**DOI:** 10.1186/s12882-023-03077-6

**Published:** 2023-02-22

**Authors:** Nida Ziauddeen, Robin F Jeffrey, Dagmar Waiblinger, Simon DS Fraser, Nisreen A Alwan, Ho M Yuen, Rafaq Azad, Dan Mason, John Wright, Richard JM Coward, Paul J Roderick

**Affiliations:** 1 School of Primary Care, Population Sciences and Medical Education, Faculty of Medicine, University of Southampton, Southampton, UK; 2 NIHR Applied Research Collaboration Wessex, Southampton, UK; 3 Bradford Institute for Health Research, Bradford Royal Infirmary, Bradford, UK; 4 NIHR Southampton Biomedical Research Centre, University of Southampton and University Hospital Southampton NHS Foundation Trust, Southampton, UK; 5 Bristol Renal, Bristol Medical School, University of Bristol, Bristol, UK

**Keywords:** foetal development, kidney volume

## Abstract

**Background:**

Foetal and early childhood development contributes to the risk of adult non-communicable diseases such as hypertension and cardiovascular disease. We aimed to investigate whether kidney size at birth is associated with markers of kidney function at 7–11 years.

**Methods:**

Foetal kidney dimensions were measured using ultrasound scans at 34 weeks gestation and used to derive kidney volume (cm^3^) in 1802 participants in the Born in Bradford (BiB) birth cohort. Blood and urine samples were taken from those who participated in the BiB follow-up at 7–11 years (n = 630) and analysed for serum creatinine, cystatin C, urea, and urinary albumin to creatinine ratio (ACR), protein to creatinine ratio (PCR) and retinol binding protein (RBP). Estimated glomerular filtration rate (eGFR) was calculated using Schwartz creatinine only and combined with cystatin C, and cystatin C only Zappitelli and Filler equations. Linear regression was used to examine the association between foetal kidney volume and eGFR, ACR, PCR and blood pressure, unadjusted and adjusted for confounders.

**Results:**

Kidney volume was positively associated in adjusted models with eGFR calculated using Schwartz combined (0.64 ml/min diff per unit increase in volume, 95% CI 0.25 to 1.02), Zappitelli (0.79, 95% CI 0.38 to 1.20) and Filler (2.84, 95% CI 1.40 to 4.28). There was an association with the presence of albuminuria but not with its level, or with other urinary markers or with blood pressure.

**Conclusion:**

Foetal kidney volume was associated with small increases in eGFR in mid-childhood. Longitudinal follow-up to investigate the relationship between kidney volume and markers of kidney function as children go through puberty is required.

**Supplementary Information:**

The online version contains supplementary material available at 10.1186/s12882-023-03077-6.

## Background

Chronic kidney disease (CKD) contributes a substantial proportion of disease burden with an estimated global prevalence of 9.1% in 2017 [1]. CKD is an independent risk factor for cardiovascular disease [2] as well as endstage kidney disease. The key to prevention of CKD and of its complications is understanding causation.

Low birthweight is associated with increased risk of subsequent adult CKD [3–5] and is also associated with adult chronic diseases associated with CKD such as hypertension and Type 2 diabetes [6–8] suggesting foetal kidney development may be a key factor. The third trimester of pregnancy is when foetal kidney development occurs and intra-uterine growth retardation (IUGR) during this period leads to reduction in nephron number [9, 10] and lower kidney size at birth [11–13]. As nephron number is fixed at birth [9], compensatory glomerular hypertrophy, hyperfiltration and hypertension, could lead to further reduction in nephrons, and susceptibility to kidney damage [14, 15].

IUGR has been found to be associated with albuminuria in infancy [16, 17], with similar estimated Glomerular Filtration Rate (eGFR) at age 2 despite smaller kidney size, suggesting hyperfiltration [17]. Kidney size tracks through into early childhood [18] and lower foetal kidney size was found to be independently associated with both reduced eGFR and kidney volume at age 6 years in the Generation R cohort in the Netherlands [19].

We aimed to investigate the relationship between foetal kidney volume and kidney function and blood pressure in childhood using the Born in Bradford (BiB) birth cohort. This is a prospective longitudinal multi-ethnic birth cohort study that aims to examine the impact of environmental, psychological and genetic factors on health and wellbeing in a deprived population. Foetal kidney volume was measured at 34 weeks gestation in a sub sample, and follow-up undertaken in childhood (age 7–11 years).

## Methods

### Baseline recruitment in pregnancy

Women were recruited to the BiB cohort study while attending for their glucose tolerance test (OGTT), offered to all pregnant women registered at Bradford Royal Infirmary at 24–28 weeks of gestation.

BiB children aged 7–11 years and their families were followed up using a multi-method approach between 2017 and 2020 (**BiB Growing Up Study**) [20]. Detailed parent and child questionnaires, BP, anthropometry and blood samples were collected. Written informed consent was collected for the follow-up and for continued routine data linkage.

### Renal ultrasound sub-study recruitment in pregnancy

A renal sub-study was nested within the full BiB cohort at baseline. Women who were attending for the OGTT at 26–28 weeks of gestation, had consented for the main BiB study and completed the baseline questionnaire, were invited to undertake a further foetal ultrasound scan (USS) at 34 weeks for standard anthropometrics and foetal renal dimension measurement. Data on renal ultrasound was available for 1802 women, details of recruitment and ultrasonography were published previously [21]. Our analyses were restricted to those who reached 37 weeks of gestation.

### Follow-up population

**Fig. 1:**
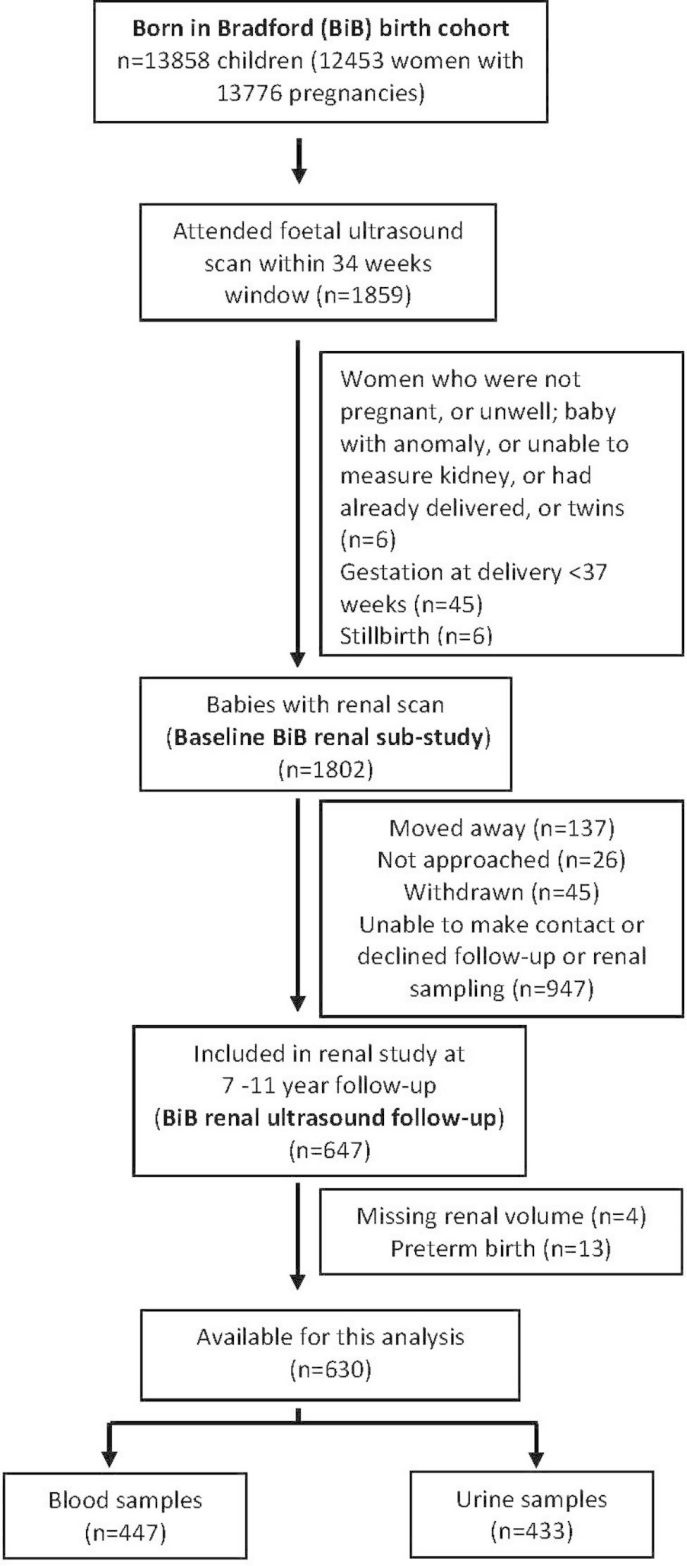
CONSORT flow chart of follow-up recruitment of the BiB renal ultrasound sub-study in pregnancy

Recruitment for the renal US sub-study follow-up commenced in January 2018 and ended in March 2020. Figure 1 shows the pattern of recruitment.

Data collection was undertaken by trained staff in schools, clinics or at the participant’s home using standard operating procedures. Child participants’ height and weight were measured without shoes and in light clothing. BP measurements were taken at the brachial artery preferably on the left arm twice using an Omron electronic monitor 705-CPII. Skinfold measurements were taken in between the first and second BP measurement. Non-fasting blood samples were taken. First morning urine samples were taken at home and transported to the laboratory at Bradford Royal Infirmary laboratory by a pre-arranged taxi service, by a parent or a member of the team.

### Laboratory methods

Blood samples were analysed for creatinine, urea and cystatin C. Creatinine and urea were analysed on Beckman AU650. The creatinine calibrator is traceable to the Isotope Dilution Mass Spectrometry (IDMS) reference method used by the National Institute of Standards and Technology Standard Reference Material 967. The coefficient of variation (CV) for serum creatinine was 5.7%. Cystatin C was measured using particle enhanced immunoturbidimetric assay on Roche/Hitachi cobas c systems. The assay is standardised against the ERM-DA471/IFCC certified reference material for cystatin C. CVs were 1.6–2.6.

Urine samples were analysed for protein, albumin, creatinine and retinol binding protein (RBP). The urine protein method was based on Pyrogallol red, urine albumin using a turbidometric assay and urine creatinine was an enzymatic assay, all measured on Beckman AU680. The limit of detection for urine albumin was 3 mg/L. CVs were 2.6% for creatinine, 8% for albumin and 7.5% for protein. RBP was measured using the immunonephelometry method on a Siemens Atellica630 Neph Nephelometer. The reference range is < 15 mg/L, the limit of detection is 3 mg/L and CV was 5.3% [22].

### Renal and BP outcomes

Outcomes included eGFR, urinary albumin to creatinine ratio (ACR), urinary protein to creatinine ratio (PCR), RBP and systolic and diastolic blood pressure. Each outcome was considered separately and all (except RBP) were assessed as continuous variables.

eGFR was calculated using four published equations as follows:

- Schwartz creatinine only = 41.3*(height (m)/ S_cr_) [23].
- 2012 CKiD Schwartz combined serum creatinine and cystatin C = 39.8 x [(height (m^2^)/S_cr_(mg/dl)]^0.456^ x [1.8/cystatin C (mg/L)]^0.418^ x [30/blood urea nitrogen (mg/dl)]^0.079^ x [1.076^male^] x [height (m)/1.4]^0.179^ [23].
- Zappittelli cystatin C only = 75.94/[cystatin C^1.17] [24].
- Filler cystatin C only log(eGFR) = 1.962 + (1.123*log(1/cystatin C)) [25].

Child height required to calculate eGFR using both Schwartz equations was measured at the same visit as the blood sample in 60.0% (n = 358), the height measurement was standardised to time of sample using centile charts if not measured at the same visit [22].

Urine albumin was only detectable in 211/416, 50.7% of the sample [22].

### Exposure

Renal volume was derived from the volume of an ellipsoid using the formula: length × width × depth × 0.523 [26].

### Covariates

#### Maternal

body mass index (BMI), age, parity, smoking, alcohol consumption, measures of socio-economic position (maternal highest educational attainment, housing tenure [buying/own house and renting or other related], and employment status), marital status and gestational diabetes (all recorded at first antenatal booking or during pregnancy).

#### Child

Birth weight, gender and gestational age at birth; age and measured weight and height at follow-up. Measured weight and height were used to calculate BMI and body surface area (BSA). BMI was converted to age- and sex- adjusted z-scores according to the UK 1990 growth reference charts. BSA was calculated using the Du Bois formula [27] (BSA = weight (kg)^0.425^ x height (cm)^0.725^ × 0.007184). Blood pressure was not identified as a covariate to adjust for from the directed acyclic graph (DAG) but as the direction of this relationship is not known, we included blood pressure as a covariate [22].

### Statistical analysis

**Fig. 2:**
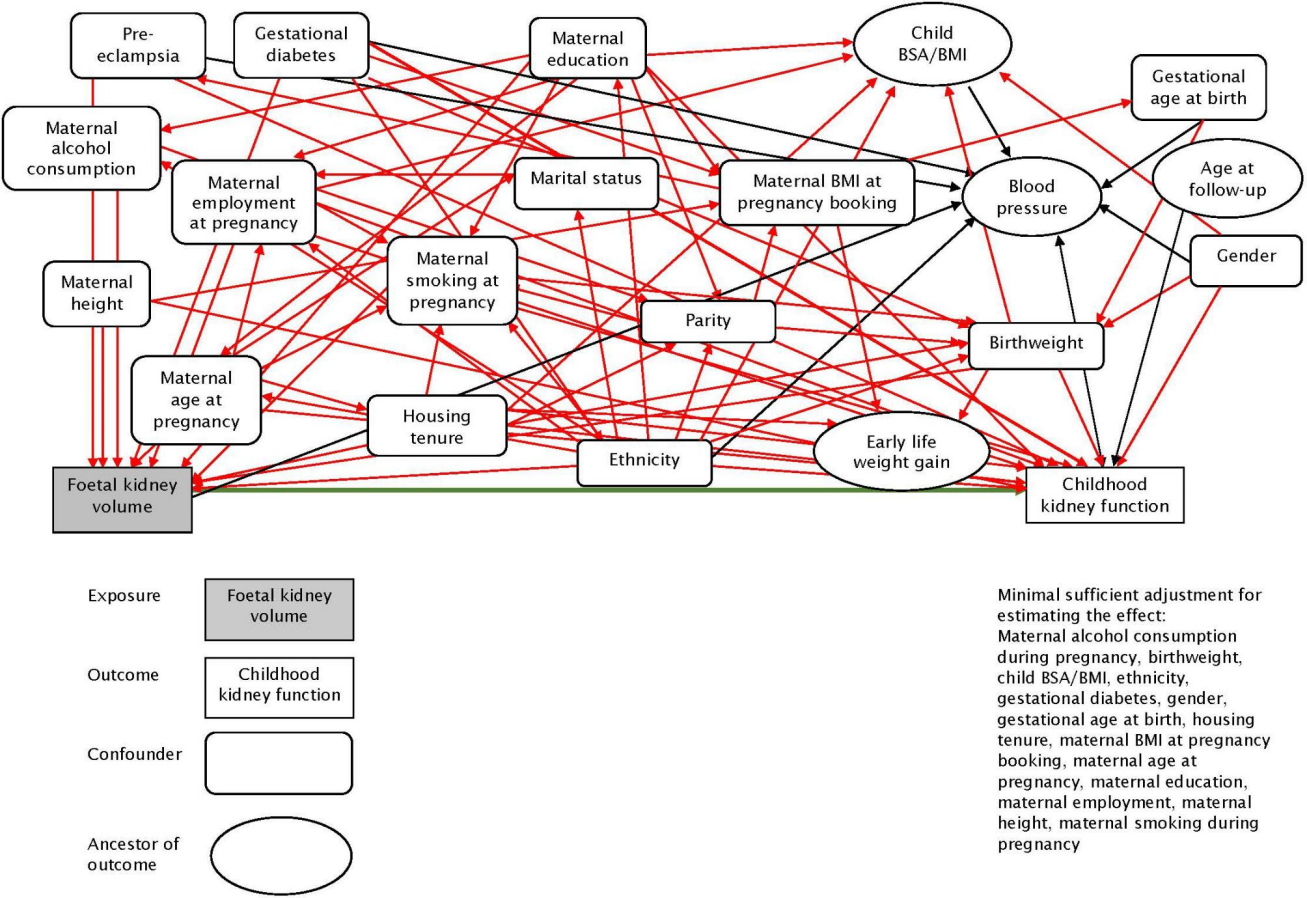
Directed acyclic graph illustrating the relationship between ethnicity and childhood kidney function

All analysis was carried out in Stata [28]. The selection of covariates into the multivariable models were guided by a DAG (Fig. 2) constructed using DAGitty [29]. The assumptions underlying the associations in the DAG were decided based on existing knowledge of the literature and topic area and through detailed discussion within the team.

Basic descriptive statistics are presented for the overall sample and stratified by tertiles of foetal renal volume. Linear regression was used to examine the association between foetal renal volume and the outcome measures, except for the ACR detectable binary outcome where logistic regression was used with the ACR result outcome conditional on a positive ACR detectable outcome. A statistical significance level of 0.01 with 95% CI was used due to the multiple comparisons performed.

Covariates were added sequentially to form multivariable regression models in stages by grouping explanatory factors:

Model 1: Ethnicity and child age at kidney function measurement (ethnicity);

Model 2: Additionally adjusted for maternal age, maternal education, housing tenure and employment status (socio-demographic);

Model 3: Additionally adjusted for maternal BMI, maternal height, smoking and alcohol consumption in pregnancy, and gestational diabetes (maternal/pregnancy);

Model 4: Further adjusted for birthweight, gestational age at birth, BSA (kidney outcomes) or BMI (BP outcomes), gender, systolic and diastolic blood pressure (kidney outcomes) (fully adjusted).

Sensitivity analysis to explore the impact of differences in timing of height measurement and blood sampling for the Schwartz eGFR formulae was undertaken. We compared estimates using standardised height (accounting for the difference in timing between sampling and measurement), height as measured (without accounting for difference in timing), and restricting the sample to those with sampling and measurement at the same time. Separate sensitivity analysis replacing BSA with BMI in eGFR models was also carried out. Complete case analysis was carried out due to the low percentage of missing data on model covariates (n < = 2.5%).

## Results

Of the 1802 children whose mothers had a renal ultrasound in pregnancy, 630 children provided a blood (n = 447) or urine (n = 433) sample for renal analysis, of which 250 children provided both a blood and urine sample (Fig. 1). The response rate was higher in South Asians and in boys but there was no difference in birthweight or renal volume between those that responded and those that did not (Table 1).

**Table 1:** Selected descriptive variables from the baseline data to assess difference in sample characteristics at follow-up by response*

	Follow-up			Non-response to renal follow-up
	Blood or urine	Blood	Urine	
	n (%)	n (%)	n (%)	n (%)
n	630	447	433	1155
Mother’s ethnic group				
White	271 (43.0)	190 (42.5)	185 (42.7)	678 (58.7)
South Asian	332 (52.7)	240 (53.7)	228 (52.7)	403 (34.9)
Other	27 (4.3)	17 (3.8)	20 (4.6)	9 (0.8)
Derived equivalized mother’s education				
None (< 5 GCSE or equivalent)	122 (19.4)	82 (18.3)	78 (18.9)	173 (15.0)
School (≥ 5 GCSE or equivalent)	182 (28.9)	137 (30.7)	118 (28.6)	377 (32.7)
Further and higher (A level or equivalent or higher)	281 (44.6)	197 (44.1)	190 (46.0)	522 (45.3)
Other (Other, Overseas, Unknown)	45 (7.1)	31 (6.9)	27 (6.5)	80 (6.9)
Gender				
Male	331 (52.5)	224 (50.1)	234 (54.0)	555 (48.1)
Female	299 (47.5)	223 (49.9)	199 (46.0)	600 (52.0)
Birthweight (mean ± SD)	3323 ± 483	3320 ± 464	3330 ± 492	3331 ± 501
Renal volume (cm^3^)	9.67 ± 2.83	9.62 ± 2.82	9.65 ± 2.86	9.75 ± 2.77
Age at renal measurement, years	8.8 ± 0.9	8.9 ± 0.9	8.8 ± 0.9	-

*Non-response were those who had a foetal renal ultrasound at baseline but did not provide a blood or urine sample for follow-up

52.7% of the sample with follow-up blood or urine sampling (n = 630) were South Asian and 44.6% had further and higher maternal education (Table 2). Mean maternal age was 28.2 years (standard deviation (SD) 5.6) and mean maternal BMI at antenatal booking was 26.7 kg/m^2^ (SD 5.9). Mothers of babies in the lowest tertile of renal volume were more likely to be of South Asian origin; to be married, and to own their own home, less likely to smoke or drink during pregnancy, and were younger, had lower BMI, and higher parity.

**Table 2:** Demographic and clinical details of mothers and children for the full sample and stratified by tertiles of fetal renal volume

Variables	Full sample			Renal volume		
	All	Blood	Urine	Tertile 1	Tertile 2	Tertile 3
n	630	447	433	210	210	210
**Mother’s details**						
Mother’s ethnic group						
White	271 (43.0)	190 (42.5)	185 (42.7)	54 (25.7)	90 (42.9)	127 (60.5)
South Asian	332 (52.7)	240 (53.7)	228 (52.7)	150 (71.4)	109 (51.9)	73 (34.8)
Other	27 (4.3)	17 (3.8)	20 (4.6)	6 (2.9)	11 (5.2)	10 (4.8)
Educational attainment						
None (< 5 GCSE or equivalent)	122 (19.4)	82 (18.3)	78 (18.0)	51 (24.3)	36 (17.1)	35 (16.7)
School (≥ 5 GCSE or equivalent)	182 (28.9)	137 (30.7)	123 (28.4)	62 (29.5)	54 (25.7)	66 (31.4)
Further (A level) and higher	281 (44.6)	197 (44.1)	204 (47.1)	86 (41.0)	106 (50.5)	89 (42.4)
Others (other, overseas, unknown)	45 (7.1)	31 (6.9)	28 (6.5)	11 (5.2)	14 (6.7)	20 (9.5)
Marital status						
Married	479 (76.0)	339 (75.8)	334 (77.1)	177 (84.3)	164 (78.1)	138 (65.7)
Single (never married, divorced or separated)	151 (24.0)	108 (24.2)	99 (22.9)	33 (15.7)	46 (21.9)	72 (34.3)
Housing tenure						
Buying/own	420 (66.7)	294 (65.8)	296 (68.4)	148 (70.5)	146 (69.5)	126 (60.0)
Renting or other related	210 (33.3)	153 (34.2)	137 (31.6)	62 (29.5)	64 (30.5)	84 (40.0)
Employment status during pregnancy						
Employed	313 (49.7)	211 (47.2)	220 (50.8)	85 (40.5)	110 (52.4)	118 (56.2)
Previously employed	167 (26.5)	135 (30.2)	102 (23.6)	56 (26.7)	54 (25.7)	57 (27.1)
Never employed	150 (23.8)	101 (22.6)	111 (25.6)	69 (32.9)	46 (21.9)	35 (16.7)
Age at antenatal booking, years	28.2 ± 5.6	28.0 ± 5.6	28.6 ± 5.6	27.6 ± 5.2	28.4 ± 5.6	28.7 ± 5.9
% missing	0.5	0.4	0.5	0.5	0.5	0.5
BMI at antenatal booking, kg/m^2^	26.7 ± 5.9	26.6 ± 5.7	26.8 ± 6.1	25.3 ± 5.1	27.0 ± 5.8	27.8 ± 6.3
% missing	2.5	2.9	2.5	2.4	3.3	1.9
Height at antenatal booking, cm	161.7 ± 6.3	161.5 ± 6.3	162.0 ± 6.3	160.2 ± 6.2	161.7 ± 6.0	163.1 ± 6.4
% missing	0.6	0.9	0.2	0.5	1.0	0.5
Parity						
0	243 (39.5)	166 (38.2)	167 (39.7)	73 (36.0)	86 (41.8)	84 (40.8)
1	177 (28.8)	131 (30.1)	117 (27.8)	53 (26.1)	60 (29.1)	64 (31.1)
2	103 (16.8)	73 (16.8)	70 (16.6)	41 (20.2)	36 (17.5)	26 (12.6)
3+	92 (15.0)	65 (15.0)	67 (15.9)	36 (18.2)	24 (12.6)	32 (15.6)
% missing	2.4	2.7	2.8	3.3	1.9	1.9
Mother smoked during pregnancy						
No	556 (88.3)	387 (86.6)	391 (90.3)	192 (91.4)	185 (88.1)	179 (85.2)
Yes	74 (11.8)	60 (13.4)	42 (9.7)	18 (8.6)	25 (11.9)	31 (14.8)
Mother drank alcohol during pregnancy						
No	549 (87.1)	389 (87.0)	375 (86.6)	196 (93.3)	178 (84.8)	175 (83.3)
Yes	81 (12.9)	58 (13.0)	58 (13.4)	14 (6.7)	32 (15.2)	35 (16.7)
Gestational diabetes						
No	581 (92.5)	418 (93.7)	396 (91.9)	196 (93.3)	197 (93.3)	190 (90.9)
Yes	47 (7.5)	28 (6.3)	35 (8.1)	14 (6.7)	14 (6.7)	19 (9.1)
% missing	0.3	0.2	0.5	-	0.5	0.5
**Renal measures (scan)**						
Volume, cm^3^	9.67 ± 2.83	9.62 ± 2.82	9.65 ± 2.86	6.91 ± 0.90	9.29 ± 0.61	12.80 ± 2.29
Volume/estimated fetal weight, cm^3^/kg	4.32 ± 1.13	4.31 ± 1.16	4.30 ± 1.11	3.26 ± 0.49	4.22 ± 0.48	5.47 ± 0.94
Volume/birth weight, cm^3^/kg	2.92 ± 0.78	2.91 ± 0.78	2.91 ± 0.76	2.24 ± 0.39	2.87 ± 0.40	3.66 ± 0.72
**Child’s**						
Gender						
Male	331 (52.5)	224 (50.1)	234 (54.0)	97 (46.2)	100 (47.6)	134 (63.8)
Female	299 (47.5)	223 (49.9)	199 (46.0)	113 (53.8)	110 (52.4)	76 (36.2)
Gestational age at birth, weeks	39.8 ± 1.1	39.9 ± 1.1	39.8 ± 1.1	39.7 ± 1.1	39.8 ± 1.2	39.9 ± 1.2
Birth weight, g	3323 ± 483	3320 ± 464	3330 ± 492	3131 ± 421	3291 ± 454	3547 ± 479
Blood pressure						
Systolic	111 ± 12	112 ± 12	111 ± 12	112 ± 11	111 ± 12	111 ± 12
Diastolic	70 ± 10	70 ± 9	70 ± 9	70 ± 9	71 ± 10	69 ± 9
% missing	4.3	2.9	5.5	5.7	1.9	5.2
Age at renal measurement, years	8.8 ± 0.9	8.9 ± 0.9	8.8 ± 0.9	8.9 ± 0.9	8.8 ± 0.9	8.8 ± 0.8
% missing	0.3	-	0.5	-	0.5	0.5
Cystatin C, mg/L						
N	426	426	239	147	141	138
Mean ± SD	0.83 ± 0.08	0.83 ± 0.08	0.83 ± 0.08	0.85 ± 0.08	0.83 ± 0.08	0.82 ± 0.08
Estimated Glomerular Filtration Rate (eGFR) (Schwartz creatinine only)*						
n	380	380	211	128	125	127
Mean ± SD	116.8 ± 17.3	116.8 ± 17.3	117.4 ± 16.9	114.5 ± 17.1	117.4 ± 16.8	118.4 ± 17.7
< 60	-	-	-	-	-	-
eGFR (Schwartz combined formula)*						
n	361	361	201	124	118	119
Mean ± SD	91.8 ± 8.8	91.8 ± 8.8	92.3 ± 8.9	89.8 ± 8.7	92.1 ± 8.0	93.7 ± 9.3
< 60	-	-	-	-	-	-
eGFR (Zappitelli CystatinC)**						
n	426	426	239	147	141	138
Mean ± SD	95.2 ± 10.3	95.2 ± 10.3	95.5 ± 10.4	93.1 ± 10.3	96.1 ± 10.1	96.6 ± 10.3
< 60	-	-	-	-	-	-
eGFR (Filler CystatinC)***						
n	426	426	239	147	141	138
Mean ± SD	153.5 ± 36.5	153.5 ± 36.5	154.3 ± 36.9	146.2 ± 36.0	156.3 ± 35.5	158.5 ± 37.1
< 60	1 (0.2)	1 (0.2)	-	-	1 (0.5)	-
Albumin creatinine ratio (ACR)						
N	416	238	416	150	127	139
Mean ± SD	0.7 ± 2.5	0.8 ± 3.2	0.7 ± 2.5	0.6 ± 1.0	0.6 ± 0.7	1.0 ± 4.2
< 2.5 (M)/<3.5 (F)	405 (97.4)	229 (96.2)	202 (97.4)	146 (97.3)	126 (99.2)	133 (95.7)
≥ 2.5 (M)/ ≥3.5 (F)	11 (2.6)	9 (3.8)	11 (2.6)	4 (2.7)	1 (0.8)	6 (4.3)
Protein creatinine ratio (PCR)						
n	392	225	392	142	117	133
Mean ± SD	10.5 ± 4.8	10.7 ± 5.8	10.5 ± 4.8	10.4 ± 3.4	10.6 ± 2.8	10.5 ± 7.1
< 15	362 (92.4)	207 (92.0)	362 (92.4)	130 (91.6)	108 (92.3)	124 (93.2)
≥ 15	30 (7.7)	18 (8.0)	30 (7.7)	12 (8.5)	9 (7.7)	9 (6.8)
Urinary retinol binding protein (RBP) (mg/L)						
< 2	429 (99.1)	247 (98.8)	429 (99.1)	155 (100)	127 (97.0)	147 (100)
3	1 (0.2)	1 (0.4)	1 (0.2)	-	1 (0.8)	-
Missing/No result	3 (0.7)	2 (0.8)	3 (0.7)	-	3 (2.3)	-

The prevalence of CKD at 7–11 years was rare (11 (2.6%) with ACR ≥ 2.5 mg/mmol in boys/3.5 mg/mmol in girls and none with eGFR < 60 ml/min/1.73m^2^). RBP results for the whole sample were within the normal range.

**Fig. 3:**
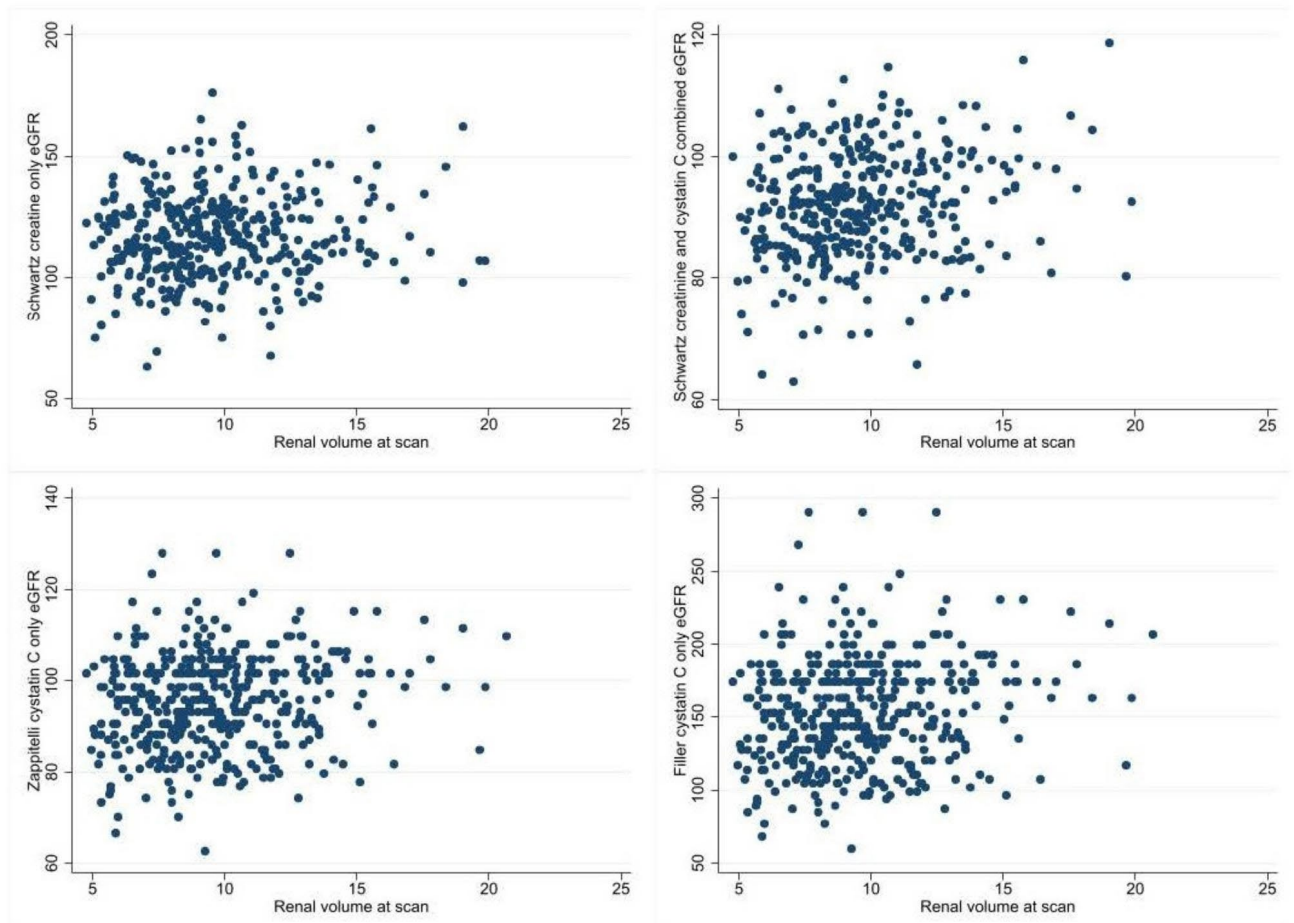
Scatter plots of eGFR and renal volume

Males were more likely to be in the highest tertile for foetal kidney volume. Foetal kidney volume was inversely associated with cystatin C and positively with eGFR on all measures. There were no obvious patterns in urinary markers or blood pressure. The correlations between renal volume and the four measures of eGFR are shown in Fig. 3.

Kidney volume was positively associated in unadjusted and adjusted models with eGFR calculated using Schwartz creatinine only formula (fully adjusted 0.80 ml/min diff per unit increase in volume, 95% CI 0.06 to 1.53), Schwartz combined (0.64 ml/min diff per unit increase in volume, 95% CI 0.25 to 1.02), Zappitelli (0.79, 95% CI 0.38 to 1.20) and Filler (2.84, 95% CI 1.40 to 4.28) (Table 3, Supplementary Table 1 for complete results of adjusted models). These findings were consistent using cystatin C as an outcome which was negatively associated with renal volume. A higher renal volume was associated with a higher probability of having a detectable ACR value but was not associated with having a higher ACR result. There were no differences in PCR, RBP or blood pressure.

**Table 3:** Summary table of differences in kidney function, kidney damage and blood pressure by foetal kidney volume: univariable and multivariable*

Model variable	Schwartz eGFR creatinine only	Schwartz eGFR combined	Zappitelli eGFR cystatin C only	Filler eGFR cystatin C only	Cystatin C	Albumin creatinine ratio		Protein creatinine ratio (logged)	Systolic blood pressure (sBP)	Diastolic blood pressure (dBP)
						Detectable	Result			
	Beta estimate (95% CI)	Beta estimate (95% CI)	Beta estimate (95% CI)	Beta estimate (95% CI)	Beta estimate (95% CI)	Odds ratio (95% CI)	Beta estimate (95% CI)	Beta estimate (95% CI)	Beta estimate (95% CI)	Beta estimate (95% CI)
**Unadjusted – n**	380	361	426	426	426	416	211	392	603	603
Renal Volume, cm^3^	0.56 -0.05 to 1.17	**0.61** **0.29 to 0.93**	**0.60** **0.25 to 0.95**	**2.12** **0.89 to 3.36**	**-0.005** **-0.007 to -0.002**	1.04 0.98 to 1.12	0.05 -0.05 to 0.15	-0.01 -0.02 to 0.004	0.004 -0.33 to 0.34	-0.02 -0.29 to 0.25
**Ethnicity - n**	380	361	426	426	426	416	211	392	603	603
Renal Volume, cm^3^	0.72 0.08 to 1.36	**0.72** **0.38 to 1.05**	**0.53** **0.17 to 0.90**	**1.89** **0.61 to 3.17**	**-0.004** **-0.007 to -0.001**	1.05 0.98 to 1.13	0.07 -0.03 to 0.17	-0.01 -0.02 to 0.004	0.13 -0.21 to 0.47	0.11 -0.16 to 0.38
**Socio-demographic - n**	380	361	426	426	426	416	211	392	603	603
Renal Volume, cm^3^	0.77 0.12 to 1.42	**0.76** **0.42 to 1.10**	**0.58** **0.21 to 0.94**	**2.05** **0.76 to 3.35**	**-0.004** **-0.007 to -0.002**	1.06 0.98 to 1.14	0.05 -0.02 to 0.11	-0.005 -0.02 to 0.01	0.08 -0.26 to 0.43	0.10 -0.18 to 0.37
**Maternal/pregnancy - n**	369	350	413	413	413	404	207	380	586	586
Renal Volume, cm^3^	0.59 -0.10 to 1.27	**0.70** **0.34 to 1.06**	**0.66** **0.29 to 1.05**	**2.37** **1.02 to 3.72**	**-0.005** **-0.008 to -0.002**	1.06 0.98 to 1.14	0.04 -0.02 to 0.11	-0.005 -0.02 to 0.01	0.02 -0.33 to 0.38	0.07 -0.21 to 0.36
**Fully adjusted - n**	355	338	386	386	386	362	182	339	553	553
Renal Volume, cm^3^	0.80 0.06 to 1.53	**0.64** **0.25 to 1.02**	**0.79** **0.38 to 1.20**	**2.84** **1.40 to 4.28**	**-0.006** **-0.009 to -0.003**	**1.14** **1.04 to 1.24**	0.08 0.01 to 0.15	0.002 -0.01 to 0.01	0.13 -0.51 to 0.24	0.03 -0.27 to 0.34

*Results presented in bold text are significant at p < 0.01

Ethnicity model is adjusted for ethnicity and child age at kidney function measurement

Socio-demographic model is ethnicity model plus maternal age, maternal educational attainment, housing tenure, and employment status, all at pregnancy

Maternal/pregnancy model is socio-demographic model plus maternal BMI, maternal height, alcohol consumption in pregnancy, maternal smoking in pregnancy, and gestational diabetes

Fully adjusted is maternal/pregnancy model plus birthweight, gestational age at birth, child body surface area (for kidney outcomes) or body mass index (for blood pressure), child gender (all outcomes), and systolic and diastolic blood pressure (for eGFR, cystatin C, ACR and PCR only)

Complete results of the Fully adjusted models is shown in Supplementary Table 1

Adjusting for child BMI instead of BSA resulted in similar estimates for eGFR (Supplementary Table 2). There was little difference in eGFR calculated using measured height regardless of timing between height measurement and blood sampling or on restricting to the subset with blood sampling and height measurement taken at the same time (Supplementary Table 3).

## Discussion

Prevention of adult CKD partly rests on better understanding of the early origins and childhood determinants of kidney function. Using a population based multi-ethnic birth cohort, we have shown here that foetal kidney volume is positively associated with eGFR using cystatin C only, and cystatin C and creatinine combined formulae and with having a detectable ACR result but not with other urinary measures or blood pressure.

### Strengths and limitations

Our study strengths include a multi-ethnic population from the same city with diverse socio-economic background with detailed ultrasonography with high reliability in pregnancy for renal measures, and first morning urine sample taken to measure albuminuria and proteinuria. Foetal kidney volume was used as a proxy for nephron number [30–32]. This is the first renal follow-up of this cohort and a key strength is the possibility of following up this cohort as the children grow. We measured both creatinine and cystatin C and calculated eGFR using four different formulae, all of which have been validated in paediatric populations. eGFR calculated using the Schwartz creatinine only formula was higher than that using the Zappitelli cystatin C as previously shown in other studies [33, 34]. It has been previously suggested that cystatin C formulae may be more sensitive than creatinine formulae for evaluating kidney function. This is because cystatin C is freely filtered by glomeruli, fully catabolised by renal tubules and not excreted by non-renal routes [35]. There was no correction for ethnicity in any of the eGFR formulae.

There were limitations. Uptake to the follow-up component was lower than expected and whilst there was no obvious major response bias this limited our statistical power. The low uptake highlights the challenge of undertaking follow-up, especially with invasive testing, in a population with high levels of socio-economic disadvantage and ethnic diversity. Differences in timing between anthropometric measurements and blood sampling in some children may have affected the creatinine based eGFR measurement. We used growth centile charts to take this into account, and no difference in study findings was found in sensitivity analysis. Our assessments of kidney function and blood pressure were based on a single measure and any variation is likely to reduce the strength of any associations but this approach was the same as in another study [36]. Unlike the Generation R cohort [30], we had no valid measures of kidney volume in childhood as this was not practically feasible. Our data are restricted to full term infants as we excluded preterm infants.

These findings add to our knowledge of the biology of kidney development by examining the influence of foetal kidney size through childhood. There are limited data on the childhood associations of kidney volume, predominantly from studies using the Generation R Dutch birth cohort. Third trimester foetal kidney volume was associated with eGFR (Schwartz creatinine based) and cystatin C at 6 years of age but there was no association with albuminuria or blood pressure in the Generation R cohort (n = 870) [37]. The effect sizes were small and without clinical consequences at school age. Foetal kidney size on ultrasound was correlated with serum creatinine at age 6 in a single centre study of 1748 children in Italy [36].

The Generation R cohort were able to extend the investigations by including ultrasound in childhood at age 6 and hence childhood kidney volume. Foetal kidney volume was positively associated with childhood kidney volume, and both were associated with eGFR and inversely with Cystatin C but not blood pressure [30, 37]. In Generation R, lower foetal growth was associated with lower childhood kidney volume and function independently of childhood growth [30]. The relationship of kidney volume with microalbuminuria is less clear. In the Generation R cohort, childhood kidney volume was positively associated with microalbuminuria and in our study foetal kidney volume was associated with the presence though not level of microalbuminuria. The significance is unclear and requires further follow-up .

Foetal kidney volume reflects nephron number which are fixed at birth. Nephron number is an important determinant of kidney function through childhood. Limited nephron number may lead to hyperfiltration and later impaired function [5]. Impaired foetal growth influences birth weight and foetal kidney size and this has persistent effects on kidney development and function and may affect adult CKD risk. Additionally, renal tissue relative to body weight may impact on eGFR. This may be part of the explanation for ethnic differences in adult CKD, as we have previously shown ethnic differences in foetal kidney volume [21].

Further longitudinal follow-up is required in the Born in Bradford cohort to investigate the relationship between foetal kidney volume, childhood measures of kidney function, blood pressure and metabolic risk with adolescent kidney function and size as children go through puberty and in later life, to see how any effects track. This would inform population strategies and risk stratification in utero/childhood to prevent adult CKD through optimising foetal and postnatal growth and kidney development. This study suggests that acting early may have advantages in reducing late-onset adult disease presentation for higher risk groups.

## Electronic supplementary material

Below is the link to the electronic supplementary material.

Supplementary Material 1

## Data Availability

Data requests can be made directly to Born in Bradford by completing an expression of. interest form available from https://borninbradford.nhs.uk/research/how-to-access-data/. and submitting it to borninbradford@bthft.nhs.uk.

## References

[1] Bikbov B, Purcell CA, Levey AS, Smith M, Abdoli A, Abebe M (2020). Global, regional, and national burden of chronic kidney disease, 1990–2017: a systematic analysis for the global burden of Disease Study 2017. The Lancet.

[2] Sarnak MJ, Levey AS, Schoolwerth AC, Coresh J, Culleton B, Hamm LL (2003). Kidney disease as a risk factor for development of cardiovascular disease: a statement from the American Heart Association councils on kidney in Cardiovascular Disease, high blood pressure research, clinical cardiology, and Epidemiology and Prevention. Circulation.

[3] White SL, Perkovic V, Cass A, Chang CL, Poulter NR, Spector T (2009). Is low Birth Weight an antecedent of CKD in later life? A systematic review of Observational Studies. Am J Kidney Dis.

[4] Silverwood RJ, Pierce M, Hardy R, Sattar N, Whincup P, Ferro C (2013). Low birth weight, later renal function, and the roles of adulthood blood pressure, diabetes, and obesity in a british birth cohort. Kidney Int.

[5] Luyckx VA, Bertram JF, Brenner BM, Fall C, Hoy WE, Ozanne SE (2013). Effect of fetal and child health on kidney development and long-term risk of hypertension and kidney disease. The Lancet.

[6] Whincup PH, Kaye SJ, Owen CG, Huxley R, Cook DG, Anazawa S (2008). Birth weight and risk of type 2 diabetes: a systematic review. JAMA.

[7] Huxley RR, Shiell AW, Law CM (2000). The role of size at birth and postnatal catch-up growth in determining systolic blood pressure: a systematic review of the literature. J Hypertens.

[8] Barker DJP, Thornburg KL (2013). The Obstetric Origins of Health for a lifetime. Clin Obstet Gynecol.

[9] Hinchliffe SA, Lynch MR, Sargent PH, Howard CV, Van Velzen D (1992). The effect of intrauterine growth retardation on the development of renal nephrons. Br J Obstet Gynaecol.

[10] Merlet-Bénichou C, Vilar J, Lelievre-Pegorier M, Moreau E, Gilbert T (1997). Fetal nephron mass: its control and deficit. Adv Nephrol Necker Hosp.

[11] Hughson M, Farris AB, Douglas-Denton R, Hoy WE, Bertram JF (2003). Glomerular number and size in autopsy kidneys: the relationship to birth weight. Kidney Int.

[12] Silver LE, Decamps PJ, Korst LM, Platt LD, Castro LC (2003). Intrauterine growth restriction is accompanied by decreased renal volume in the human fetus. Am J Obstet Gynecol.

[13] Konje JC, Bell SC, Morton JJ, de Chazal R, Taylor DJ (1996). Human fetal kidney morphometry during gestation and the relationship between weight, kidney morphometry and plasma active renin concentration at birth. Clin Sci Lond Engl 1979.

[14] Brenner BM, Chertow GM (1994). Congenital oligonephropathy and the etiology of adult hypertension and progressive renal injury. Am J Kidney Dis Off J Natl Kidney Found.

[15] Zohdi V, Sutherland MR, Lim K, Gubhaju L, Zimanyi MA, Black MJ (2012). Low Birth Weight due to Intrauterine Growth Restriction and/or Preterm Birth: Effects on Nephron Number and Long-Term Renal Health. Int J Nephrol.

[16] Zanardo V, Fanelli T, Weiner G, Fanos V, Zaninotto M, Visentin S (2011). Intrauterine growth restriction is associated with persistent aortic wall thickening and glomerular proteinuria during infancy. Kidney Int.

[17] Iyengar A, Nesargi S, George A, Sinha N, Selvam S, Luyckx VA (2016). Are low birth weight neonates at risk for suboptimal renal growth and function during infancy?. BMC Nephrol.

[18] Geelhoed JJM, Verburg BO, Nauta J, Lequin M, Hofman A, Moll HA (2009). Tracking and determinants of kidney size from fetal life until the age of 2 years: the Generation R Study. Am J Kidney Dis Off J Natl Kidney Found.

[19] Bakker H, Gaillard R, Franco OH, Hofman A, van der Heijden AJ, Steegers EAP (2014). Fetal and infant growth patterns and kidney function at school age. J Am Soc Nephrol JASN.

[20] Bird PK, McEachan RRC, Mon-Williams M, Small N, West J, Whincup P (2019). Growing up in Bradford: protocol for the age 7–11 follow up of the born in Bradford birth cohort. BMC Public Health.

[21] Roderick PJ, Jeffrey RF, Yuen HM, Godfrey KM, West J, Wright J (2016). Smaller kidney size at birth in South Asians: findings from the born in Bradford birth cohort study. Nephrol Dial Transplant.

[22] Ziauddeen N, Jeffrey RF, Waiblinger D, Fraser SDS, Alwan NA, Yuen HM (2022). Ethnic differences in kidney function in childhood: the born in Bradford Cohort Renal Study. Wellcome Open Res.

[23] Schwartz GJ, Muñoz A, Schneider MF, Mak RH, Kaskel F, Warady BA (2009). New equations to estimate GFR in children with CKD. J Am Soc Nephrol JASN.

[24] Zappitelli M, Parvex P, Joseph L, Paradis G, Grey V, Lau S (2006). Derivation and validation of cystatin C-based prediction equations for GFR in children. Am J Kidney Dis Off J Natl Kidney Found.

[25] Filler G, Lepage N (2003). Should the Schwartz formula for estimation of GFR be replaced by cystatin C formula?. Pediatr Nephrol.

[26] Chitty LS, Altman DG (2003). Charts of fetal size: kidney and renal pelvis measurements. Prenat Diagn.

[27] Du Bois D, Du Bois EF. A formula to estimate the approximate surface area if height and weight be known. 1916. Nutr Burbank Los Angel Cty Calif 1989;5:303–11; discussion 312–313. 2520314

[28] Stata Statistical Software. : Release 17 2021.

[29] Textor J, van der Zander B, Gilthorpe MS, Liskiewicz M, Ellison GT (2016). Robust causal inference using directed acyclic graphs: the R package “dagitty.”. Int J Epidemiol.

[30] Bakker H, Kooijman MN, van der Heijden AJ, Hofman A, Franco OH, Taal HR (2014). Kidney size and function in a multi-ethnic population-based cohort of school-age children. Pediatr Nephrol.

[31] Keller G, Zimmer G, Mall G, Ritz E, Amann K (2003). Nephron number in patients with primary hypertension. N Engl J Med.

[32] Mañalich R, Reyes L, Herrera M, Melendi C, Fundora I (2000). Relationship between weight at birth and the number and size of renal glomeruli in humans: a histomorphometric study. Kidney Int.

[33] Bacchetta J, Cochat P, Rognant N, Ranchin B, Hadj-Aissa A, Dubourg L (2011). Which creatinine and cystatin C equations can be reliably used in children?. Clin J Am Soc Nephrol CJASN.

[34] Bakker H, Gaillard R, Hofman A, Reiss IK, Steegers EAP, Jaddoe VWV (2017). Fetal first trimester growth is not associated with kidney outcomes in childhood. Pediatr Nephrol.

[35] Andersen TB, Eskild-Jensen A, Frøkiær J, Brøchner-Mortensen J (2009). Measuring glomerular filtration rate in children; can cystatin C replace established methods? A review. Pediatr Nephrol.

[36] Di Zazzo G, Stringini G, Matteucci MC, Muraca M, Malena S, Emma F (2011). Serum creatinine levels are significantly influenced by renal size in the normal pediatric population. Clin J Am Soc Nephrol CJASN.

[37] Kooijman MN, Bakker H, van der Heijden AJ, Hofman A, Franco OH, Steegers EAP (2014). Childhood kidney outcomes in relation to fetal blood Flow and kidney size. J Am Soc Nephrol.

