# Supplementary material for: Role of foetal kidney size on kidney function in childhood: the born in bradford cohort renal study
Source: BMC Nephrol. 2023 Feb 22;24:41. doi: 10.1186/s12882-023-03077-6 (PMC9945391; doi:10.1186/s12882-023-03077-6)
Supplement: Supplementary file 1 — Supplementary Material 1 [file 12882_2023_3077_MOESM1_ESM.docx]

Supplementary Table 1: Summary table of covariates in multivariable analysis of differences in kidney function, kidney damage and blood pressure by renal volume

| Model variable | Schwartz eGFR creatinine | Schwartz eGFR combined | Zappitelli eGFR cystatin C | Filler eGFR cystatin C only | Cystatin C | ACR* | | PCR (logged) | Systolic blood pressure | Diastolic blood pressure |
| --- | --- | --- | --- | --- | --- | --- | --- | --- | --- | --- |
|  |  |  |  |  |  | Detectable | Result |  |  |  |
|  | Beta estimate (95% CI) | Beta estimate (95% CI) | Beta estimate (95% CI) | Beta estimate (95% CI) | Beta estimate (95% CI) | Odds ratio (95% CI) | Beta estimate (95% CI) | Beta estimate (95% CI) | Beta estimate (95% CI) | Beta estimate (95% CI) |
| **Fully adjusted model – n** | 355 | 338 | 386 | 386 | 386 | 362 | 182 | 339 | 553 | 553 |
| Renal Volume, cm^3^ | 0.80  0.06 to 1.53 | **0.64**  **0.25 to 1.02** | **0.79**  **0.38 to 1.20** | **2.84**  **1.40 to 4.28** | **-0.006**  **-0.009 to -0.003** | **1.14**  **1.04 to 1.24** | 0.08  0.01 to 0.15 | 0.002  -0.01 to 0.01 | 0.13  -0.51 to 0.24 | 0.03  -0.27 to 0.34 |
| **Covariates** |  |  |  |  |  |  |  |  |  |  |
| Ethnicity |  |  |  |  |  |  |  |  |  |  |
| White | Ref | Ref | Ref | Ref | Ref | Ref | Ref | Ref | Ref | Ref |
| South Asian | 5.87  0.59 to 11.15 | 2.49  -0.30 to 5.27 | 0.13  -2.82 to 3.08 | 0.33  -10.12 to 10.78 | -0.002  -0.02 to 0.02 | 0.89  0.46 to 1.71 | 0.09  -0.15 to 0.33 | 0.03  -0.06 to 0.12 | 0.10  -2.61 to 2.81 | 2.22  0.03 to 4.41 |
| Other | -8.21  -18.00 to 1.59 | -2.09  -7.11 to 2.93 | 5.18  -0.32 to 10.67 | 20.70  1.24 to 40.17 | -0.03  -0.07 to 0.01 | 0.46  0.15 to 1.41 | 0.38  -0.36 to 1.12 | 0.01  -0.14 to 0.17 | -0.53  -5.38 to 4.32 | 1.57  -2.35 to 5.48 |
| Maternal age at baseline, years | -0.09  -0.46 to 0.28 | -0.10  -0.30 to 0.09 | -0.17  -0.37 to 0.04 | -0.56  -1.30 to 0.17 | 0.001  -0.0002 to 0.003 | 0.98  0.94 to 1.03 | -0.004  -0.03 to 0.02 | -0.002  -0.008 to 0.005 | 0.13  -0.06 to 0.33 | 0.02  -0.14 to 0.18 |
| Derived equivalized mother’s education |  |  |  |  |  |  |  |  |  |  |
| None (<5 GCSE or equivalent) | Ref | Ref | Ref | Ref | Ref | Ref | Ref | Ref | Ref | Ref |
| School (≥5 GCSE or equivalent) | 3.93  -1.39 to 9.26 | 1.92  -0.87 to 4.71 | 1.90  -1.13 to 4.94 | 5.96  -4.80 to 16.71 | -0.02  -0.04 to 0.01 | 1.04  0.49 to 2.22 | **0.66**  **0.25 to 1.07** | 0.04  -0.06 to 0.14 | 1.29  -1.63 to 4.20 | 1.38  -0.98 to 3.74 |
| Further (A level or equivalent) and higher | 4.19  -1.24 to 9.62 | 2.55  -0.28 to 5.37 | 3.51  0.47 to 6.55 | 11.47  0.68 to 22.25 | -0.03  -0.05 to -0.01 | 1.08  0.52 to 2.22 | 0.11  -0.18 to 0.40 | 0.04  -0.06 to 0.13 | -0.25  -3.14 to 2.64 | -0.02  -2.36 to 2.31 |
| Other (Other, Overseas, Unknown) | -0.03  -8.62 to 8.56 | -0.31  -4.80 to 4.19 | 1.82  -2.98 to 6.62 | 4.89  -12.11 to 21.89 | -0.02  -0.06 to 0.02 | 2.15  0.75 to 6.18 | 0.01  -0.36 to 0.38 | -0.03  -0.13 to 0.18 | 3.53  -0.87 to 7.94 | 2.15  -1.14 to 5.71 |
| Housing tenure |  |  |  |  |  |  |  |  |  |  |
| Buying/own | Ref | Ref | Ref | Ref | Ref | Ref | Ref | Ref | Ref | Ref |
| Renting or other related | -2.67  -6.77 to 1.44 | -1.60  -3.77 to 0.57 | -2.67  -5.00 to -0.33 | -9.70  -17.98 to -1.42 | 0.02  0.001 to 0.04 | 0.74  0.44 to 1.24 | -0.17  -0.38 to 0.05 | -0.08  -0.15 to -0.01 | -0.09  -2.24 to 2.07 | -0.83  -2.57 to 0.91 |
| Employment status during pregnancy |  |  |  |  |  |  |  |  |  |  |
| Currently employed | Ref | Ref | Ref | Ref | Ref | Ref | Ref | Ref | Ref | Ref |
| Previously employed | -1.70  -6.45 to 3.04 | -0.61  -3.14 to 1.92 | 0.04  -2.66 to 2.74 | 0.67  -8.89 to 10.24 | 0.002  -0.02 to 0.02 | 0.75  0.40 to 1.39 | -0.05  -0.28 to 0.19 | 0.03  -0.06 to 0.11 | 0.52  -2.00 to 3.05 | -1.38  -3.42 to 0.66 |
| Never employed | 1.59  -4.02 to 7.20 | 0.89  -2.08 to 3.86 | 0.56  -2.63 to 3.75 | 1.98  -9.33 to 13.29 | -0.004  -0.03 to 0.02 | 0.91  0.45 to 1.81 | 0.34  -0.02 to 0.70 | 0.06  -0.03 to 0.16 | 0.91  -2.08 to 3.91 | -1.92  -4.35 to 0.50 |
| Maternal BMI at booking (kg/m^2^) | 0.22  -0.15 to 0.58 | 0.02  -0.17 to 0.21 | -0.11  -0.31 to 0.08 | -0.43  -1.11 to 0.25 | 0.001  -0.001 to 0.002 | 1.00  0.96 to 1.04 | 0.01  -0.004 to 0.03 | 0.003  -0.002 to 0.009 | -0.03  -0.21 to 0.15 | 0.02  -0.12 to 0.17 |
| Maternal height at booking (cm) | 0.15  -0.17 to 0.47 | 0.08  -0.09 to 0.25 | -0.001  -0.18 to 0.18 | -0.02  -0.66 to 0.62 | 0.000  -0.001 to 0.001 | 0.99  0.95 to 1.03 | 0.01  -0.007 to 0.03 | 0.004  -0.001 to 0.009 | 0.04  -0.12 to 0.21 | -0.03  -0.17 to 0.10 |
| Drank alcohol at any time during pregnancy |  |  |  |  |  |  |  |  |  |  |
| No | Ref | Ref | Ref | Ref | Ref | Ref | Ref | Ref | Ref | Ref |
| Yes | -1.16  -7.18 to 4.86 | -0.32  -3.53 to 2.90 | -0.57  -3.88 to 2.74 | -1.82  -13.55 to 9.91 | 0.005  -0.02 to 0.03 | 0.51  0.24 to 1.08 | 0.33  -0.08 to 0.75 | 0.03  -0.07 to 0.13 | -1.62  -4.71 to 1.47 | -0.43  -2.93 to 2.07 |
| Smoked at any time during pregnancy |  |  |  |  |  |  |  |  |  |  |
| No | Ref | Ref | Ref | Ref | Ref | Ref | Ref | Ref | Ref | Ref |
| Yes | 5.52  -0.56 to 11.59 | 2.13  -1.04 to 5.29 | 0.03  -3.36 to 3.42 | -0.34  -12.35 to 11.67 | -0.002  -0.03 to 0.02 | 1.26  0.52 to 3.06 | -0.45  -0.81 to 0.09 | -0.04  -0.15 to 0.08 | 0.65  -2.60 to 3.91 | 1.50  -1.13 to 4.13 |
| Gestational diabetes |  |  |  |  |  |  |  |  |  |  |
| No | Ref | Ref | Ref | Ref | Ref | Ref | Ref | Ref | Ref | Ref |
| Yes | 3.90  -4.24 to 12.05 | 0.38  -3.80 to 4.56 | 0.75  -3.76 to 5.25 | 1.97  -13.99 to 17.94 | -0.01  -0.04 to 0.03 | 3.49  1.23 to 9.89 | -0.04  -0.33 to 0.25 | -0.07  -0.20 to 0.06 | -2.42  -6.55 to 1.71 | 0.52  -2.82 to 3.86 |
| Child gender |  |  |  |  |  |  |  |  |  |  |
| Male | Ref | Ref | Ref | Ref | Ref | Ref | Ref | Ref | Ref | Ref |
| Female | 4.99  1.18 to 8.80 | **-3.03**  **-5.04 to -1.02** | 2.53  0.43 to 4.63 | 9.03  1.59 to 16.46 | -0.02  -0.03 to -0.003 | **2.18**  **1.37 to 3.46** | 0.07  -0.20 to 0.35 | 0.08  0.02 to 0.14 | 0.37  -1.58 to 2.31 | 0.93  -0.64 to 2.51 |
| Gestational age at birth, weeks | -0.33  -2.26 to 1.59 | -0.60  -1.61 to 0.41 | -0.90  -1.94 to 0.14 | -3.20  -6.89 to 0.48 | 0.01  -0.001 to 0.015 | 1.13  0.90 to 1.43 | 0.12  -0.02 to 0.27 | 0.01  -0.02 to 0.04 | -0.76  -1.73 to 0.22 | -0.02  -0.81 to 0.77 |
| Birthweight, g | -0.003  -0.008 to 0.002 | -0.0003  -0.003 to 0.002 | 0.001  -0.001 to 0.004 | 0.005  -0.005 to 0.014 | -0.00001  -0.00003 to 0.00001 | 1.00  1.00 to 1.00 | -0.001  -0.001 to -0.0001 | 0.000  0.000 to 0.000 | 0.002  -0.001 to 0.004 | 0.0001  -0.002 to 0.002 |
| Body surface area, m^2^ | 3.65  -10.05 to 17.34 | 0.02  -7.14 to 7.18 | **-14.37**  **-22.09 to -6.66** | **-51.00**  **-78.33 to -23.68** | **0.11**  **0.05 to 0.17** | 0.09  0.01 to 0.58 | **-1.00**  **-1.72 to -0.27** | **-0.33**  **-0.57 to -0.10** |  |  |
| Child BMI at kidney function measurement, z-score |  |  |  |  |  |  |  |  |  |  |
| Underweight |  |  |  |  |  |  |  |  | **-7.31**  **-12.05 to -2.56** | -4.05  -7.89 to -0.22 |
| Normal weight |  |  |  |  |  |  |  |  | Ref | Ref |
| Overweight/obese |  |  |  |  |  |  |  |  | **4.19**  **1.96 to 6.42** | **2.94**  **1.13 to 4.74** |
| Blood pressure |  |  |  |  |  |  |  |  |  |  |
| Systolic | 0.07  -0.13 to 0.28 | 0.03  -0.08 to 0.14 | -0.004  -0.12 to 0.11 | -0.02  -0.42 to 0.39 | 0.000  -0.001 to 0.001 | 1.03  1.01 to 1.06 | 0.004  -0.01 to 0.02 | 0.001  -0.002 to 0.004 |  |  |
| Diastolic | 0.04  -0.21 to 0.29 | -0.04  -0.17 to 0.09 | -0.12  -0.26 to 0.02 | -0.41  -0.89 to 0.08 | 0.001  0.000 to 0.002 | 0.96  0.93 to 0.99 | 0.001  -0.01 to 0.01 | 0.001  -0.003 to 0.005 |  |  |
| Child age at kidney function measurement, months | -0.12  -0.32 to 0.09 | -0.001  -0.11 to 0.11 | -0.02  -0.13 to 0.10 | -0.05  -0.45 to 0.35 | 0.0002  -0.001 to 0.001 | 1.02  0.99 to 1.05 | 0.01  -0.01 to 0.02 | **-0.01**  **-0.01 to -0.002** | **0.30**  **0.22 to 0.38** | **0.21**  **0.15 to 0.27** |

Supplementary Table 2: Summary table of differences in kidney function, kidney damage and blood pressure by ethnicity: univariable and multivariable adjusting for child BMI (not body surface area)

| Model variable | Schwartz eGFR creatinine only equation | Schwartz eGFR combined equation | Zappitelli eGFR cystatin C only equation | Filler eGFR cystatin C only equation | Cystatin C | ACR* | | PCR (logged) |
| --- | --- | --- | --- | --- | --- | --- | --- | --- |
|  |  |  |  |  |  | Detectable | Result |  |
|  | Beta estimate (95% CI) | Beta estimate (95% CI) | Beta estimate (95% CI) | Beta estimate (95% CI) | Beta estimate (95% CI) | Odds ratio (95% CI) | Beta estimate (95% CI) | Beta estimate (95% CI) |
| **Unadjusted – n** | 380 | 361 | 426 | 426 | 426 | 416 | 211 | 392 |
| Renal Volume, cm^3^ | 0.56  -0.05 to 1.17 | **0.61**  **0.29 to 0.93** | **0.60**  **0.25 to 0.95** | **2.12**  **0.89 to 3.36** | **-0.005**  **-0.007 to -0.002** | 1.04  0.98 to 1.12 | 0.05  -0.05 to 0.15 | -0.01  -0.02 to 0.004 |
| **Ethnicity adjusted - n** | 380 | 361 | 426 | 426 | 426 | 416 | 211 | 392 |
| Renal Volume, cm^3^ | 0.72  0.08 to 1.36 | **0.72**  **0.38 to 1.05** | **0.53**  **0.17 to 0.90** | **1.89**  **0.61 to 3.17** | **-0.004**  **-0.007 to -0.001** | 1.05  0.98 to 1.13 | 0.07  -0.03 to 0.17 | -0.01  -0.02 to 0.004 |
| **Socio-demographic - n** | 380 | 361 | 426 | 426 | 426 | 416 | 211 | 392 |
| Renal Volume, cm^3^ | 0.77  0.12 to 1.42 | **0.76**  **0.42 to 1.10** | **0.58**  **0.21 to 0.94** | **2.05**  **0.76 to 3.35** | **-0.004**  **-0.007 to -0.002** | 1.06  0.98 to 1.14 | 0.05  -0.02 to 0.11 | -0.005  -0.02 to 0.01 |
| **Maternal/pregnancy - n** | 369 | 350 | 413 | 413 | 413 | 404 | 207 | 380 |
| Renal Volume, cm^3^ | 0.59  -0.10 to 1.27 | **0.70**  **0.34 to 1.06** | **0.66**  **0.29 to 1.05** | **2.37**  **1.02 to 3.72** | **-0.005**  **-0.008 to -0.002** | 1.06  0.98 to 1.14 | 0.04  -0.02 to 0.11 | -0.005  -0.02 to 0.01 |
| **Fully adjusted - n** | 355 | 338 | 386 | 386 | 386 | 362 | 182 | 339 |
| Renal Volume, cm^3^ | 0.88  0.15 to 1.61 | **0.67**  **0.29 to 1.05** | **0.73**  **0.32 to 1.13** | **2.61**  **1.16 to 4.06** | **-0.005**  **-0.009 to -0.002** | **1.13**  **1.03 to 1.23** | 0.08  0.01 to 0.15 | 0.001  -0.01 to 0.01 |

Ethnicity model is adjusted for ethnicity and child age at kidney function measurement

Socio-demographic model is ethnicity model plus maternal age, maternal educational attainment, housing tenure, and employment status, all at pregnancy

Maternal/pregnancy model is socio-demographic model plus maternal BMI, maternal height, alcohol consumption in pregnancy, maternal smoking in pregnancy, and gestational diabetes

Fully adjusted is maternal/pregnancy model plus birthweight, gestational age at birth, child body mass index, child gender, and systolic and diastolic blood pressure.

Supplementary table 3: Sensitivity analysis to explore impact of differences in timing of height measurement and blood sampling for the Schwartz eGFR formulae

|  | Schwartz creatinine only formula | | | Schwartz combined formula | | |
| --- | --- | --- | --- | --- | --- | --- |
|  | Centile adjusted height | Height as measured | | Centile adjusted height | Height as measured | |
|  |  | Full sample | Sample with height measured and blood sampled at same time |  | Full sample | Sample with height measured and blood sampled at same time |
| **Mean eGFR** | 116.8 ± 17.3 | 116.0 ± 17.3 | 116.7 ± 17.6 | 91.8 ± 8.8 | 91.5 ± 8.9 | 91.7 ± 9.1 |
| **Unadjusted – n** | 380 | 380 | 257 | 361 | 361 | 249 |
| Renal Volume, cm^3^ | 0.56  -0.05 to 1.17 | 0.52  -0.09 to 1.13 | 0.69  -0.05 to 1.42 | **0.61**  **0.29 to 0.93** | **0.59**  **0.27 to 0.91** | **0.70**  **0.31 to 1.09** |
| **Ethnicity adjusted - n** | 380 | 380 | 257 | 361 | 361 | 249 |
| Renal Volume, cm^3^ | 0.72  0.08 to 1.36 | 0.68  0.04 to 1.33 | 0.83  0.04 to 1.62 | **0.72**  **0.38 to 1.05** | **0.70**  **0.37 to 1.04** | **0.80**  **0.39 to 1.22** |
| **Socio-demographic model - n** | 380 | 380 | 257 | 361 | 361 | 249 |
| Renal Volume, cm^3^ | 0.77  0.12 to 1.42 | 0.73  0.07 to 1.38 | 0.87  0.08 to 1.66 | **0.76**  **0.42 to 1.10** | **0.74**  **0.40 to 1.08** | **0.80**  **0.38 to 1.22** |
| **Maternal/pregnancy model – n** | 369 | 369 | 252 | 350 | 350 | 244 |
| Renal Volume, cm^3^ | 0.59  -0.10 to 1.27 | 0.54  -0.14 to 1.23 | 0.77  -0.07 to 1.60 | **0.70**  **0.34 to 1.06** | **0.68**  **0.32 to 1.04** | **0.78**  **0.34 to 1.22** |
| **Fully adjusted - n** | 355 | 355 | 245 | 338 | 338 | 237 |
| Renal Volume, cm^3^ | 0.80  0.06 to 1.53 | 0.73  -0.01 to 1.47 | 0.80  -0.12 to 1.71 | **0.64**  **0.25 to 1.02** | **0.61**  **0.22 to 1.00** | **0.70**  **0.23 to 1.17** |

Ethnicity model is adjusted for ethnicity and child age at kidney function measurement

Socio-demographic model is ethnicity model plus maternal age, maternal educational attainment, housing tenure, and employment status, all at pregnancy

Maternal/pregnancy model is socio-demographic model plus maternal BMI, maternal height, alcohol consumption in pregnancy, maternal smoking in pregnancy, and gestational diabetes

Fully adjusted is maternal/pregnancy model plus birthweight, gestational age at birth, child body surface area, child gender, and systolic and diastolic blood pressure.
